# A Rare Etiology of Cystic Lung Disease

**DOI:** 10.7759/cureus.24334

**Published:** 2022-04-21

**Authors:** . Parul, Pawan K Singh

**Affiliations:** 1 Pulmonary and Critical Care Medicine, Pandit Bhagwat Dayal Sharma Post Graduate Institute of Medical Sciences (PGIMS), Rohtak, IND; 2 Pulmonary and Critical Care Medicine, Pandit Bhagwat Dayal Sharma Post Graduate Institute Of Medical Sciences (PGIMS), Rohtak, IND

**Keywords:** pancreatic adenocarcinoma, endoscopic biopsy, lung metastasis, cystic lesions of the lung, pancreatic malignancy

## Abstract

Metastases presenting in the form of cystic lung disease are a very rare occurrence. Even rarer is the association of such metastases with primary pancreatic carcinoma. Here we have described the case of a 60-year-old man who presented with a six-month history of worsening shortness of breath and dry cough associated with loss of weight and appetite. At the time of admission, his oxygen saturation on room air was found to be 82% and the respiratory rate was 28/minute. Apart from bilateral diffuse coarse crackles, the rest of the physical examination was largely unremarkable. All his blood tests were found to be normal except for raised levels of alkaline phosphatase (268 IU/L). The levels of cancer antigen (CA) 19-9 were also found to be elevated (238.8 IU/ml). CT of the chest and abdomen revealed the presence of multiple cystic lesions with predominance in the lower lobes of the lungs and an enlarged head of pancreas. The patient underwent side-view esophagogastroduodenoscopy and multiple biopsies taken from the lesion in the head of pancreas revealed the presence of multiple atypical cells with glandular formation suggestive of adenocarcinoma. We were not able to directly demonstrate the tumor cells in lung tissue, as the patient was not fit for bronchoscopic procedures due to high oxygen requirements. However, the temporal course of the development of symptoms, and cysts do correlate suggesting metastasis as the etiology of cystic lesions found in his lung.

## Introduction

Although pulmonary metastases from extra-thoracic malignancies are quite common, very rarely do they present in the form of cystic lesions. Cystic metastases in lungs associated with pancreatic carcinoma have only been described once in literature [1]. Regardless of the cause of cysts, patients usually present with either no symptoms, with the cysts discovered on chest imaging done for some other reason, or with nonspecific symptoms such as cough and shortness of breath [2]. Non-specific presentation and their low incidence can lead to missing out on the diagnosis, which is also aided by the fact that such patients usually do not undergo extensive radiographic imaging till very late stages of illness. Here we describe a case of a 60-year-old man presenting with cystic pulmonary metastases that were later found to be due to primary pancreatic adenocarcinoma.

## Case presentation

**Figure 1:**
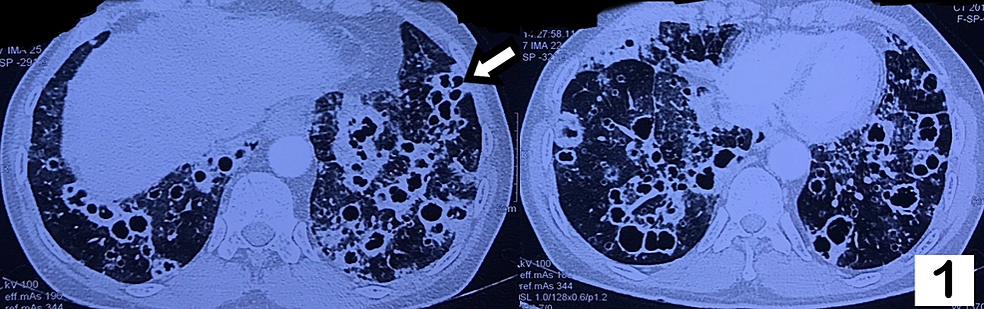
High-resolution CT of thorax showing multiple thin-walled, variable-sized, lower lobe predominant air-containing spaces. Arrow indicates air-filled cysts in the lung parenchyma. Accompanying the cysts, are randomly distributed nodules.

**Figure 2:**
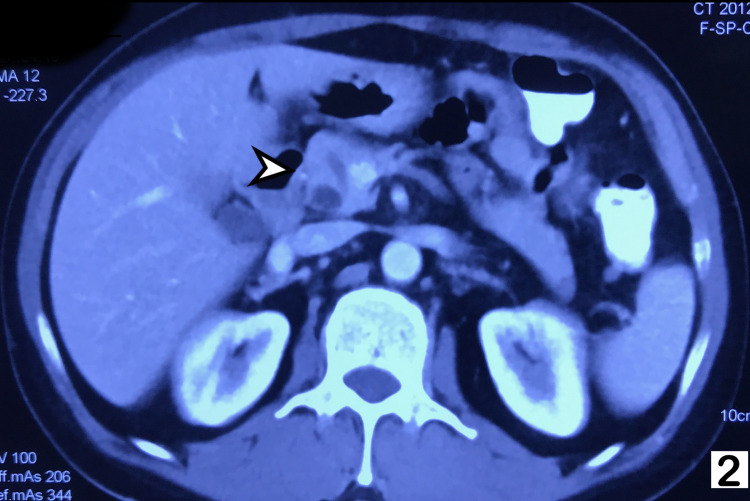
Contrast-enhanced CT of abdomen showing enlarged head of pancreas (indicated by the arrow) with dilated main pancreatic duct.

A 60-year-old male presented with progressively worsening shortness of breath and dry cough for the past six months. There was a significant history of loss of appetite and weight (unquantified). He was a heavy smoker and daily alcohol consumer. He was variably treated with bronchodilator therapy with no response. Before his illness started, his routine chest radiograph was largely unremarkable (nine months ago). At the time of admission, his room air saturation was 82%, and his respiratory rate was 28/minute. His other vitals and systemic examination were largely unremarkable except for bilateral diffuse coarse crackles. Complete blood count, renal function tests, and liver function tests were within normal limits except for the raised alkaline phosphatase (268 IU/Liter). After his chest radiograph, he underwent contrast-enhanced chest and abdomen CT scans. Findings in the scan revealed the presence of multiple cystic lesions in the lungs with predominance in the lower lobes along with the enlarged head of the pancreas with dilated main pancreatic duct (Figure 1 and Figure 2).

**Figure 3:**
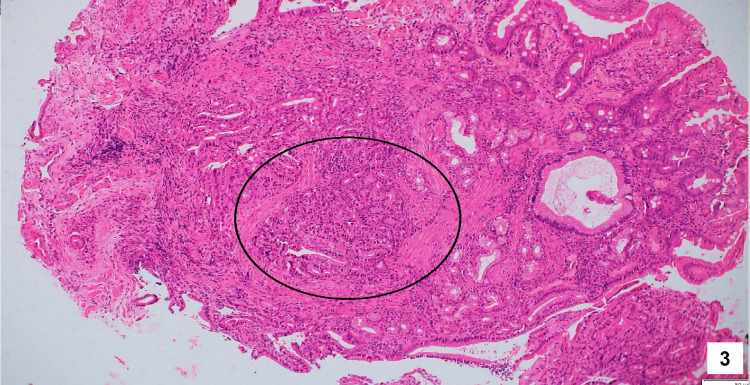
Hematoxylin and eosin staining of the biopsy tissue obtained from the bulky and friable papillae showing microscopic foci of atypical cells with glandular formation, detachment from the lining epithelium, and occasional mitosis.

**Figure 4:**
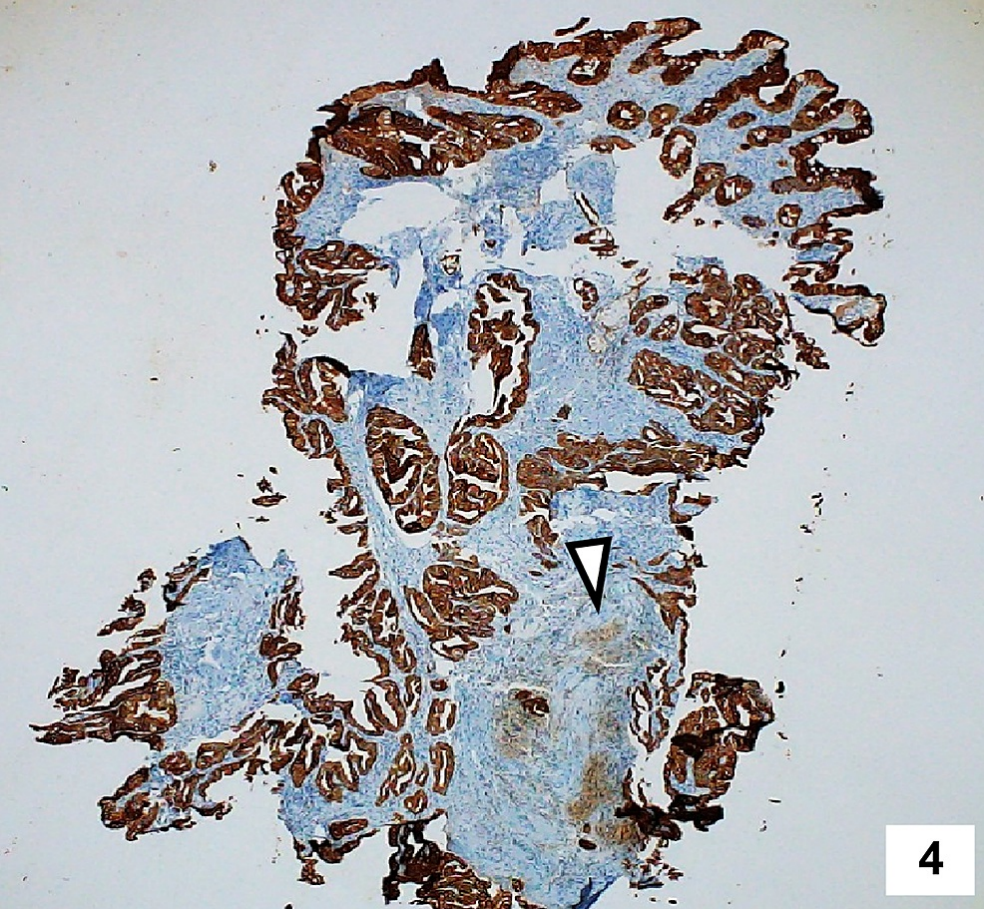
Immunohistochemistry of the biopsy specimen showing diffuse positivity for CK-7 suggesting pancreatic ductal adenocarcinoma.

His makers of malignancy showed elevated cancer antigen (CA) 19-9 (238.8IU/ml). For the evaluation of the same and bulky head of pancreas, he underwent side-view esophagogastroduodenoscopy (EGD), where he was found to have congested, and bulky papillae with friable mucosa that bled to the touch. From the same lesion, multiple biopsies were taken. The biopsy revealed the presence of atypical cells with glandular formation suggestive of adenocarcinoma (Figure 3 and Figure 4).

In view of metastatic disease (multiple bilateral pulmonary cystic metastasis) and poor performance status (European co-operative oncology group status IV), the patient was given supportive management during hospital admission, including oxygen supplementation, pain, and anxiety relief, and deep vein thrombus prophylaxis. His condition and type of disease were explained to the family. Given his poor performance status and general condition, he was continued on best supportive care, and eventually over the next three to four days, he succumbed to his illness due to progressive respiratory failure. 

## Discussion

Cyst in high-resolution CT of the thorax represents an air-filled space with discernible thin walls. It can be confused with emphysema, cavity, bullae, bleb, or bronchiectasis. There are several differential diagnoses for cystic lung disease like lymphangioleiomyomatosis, lymphocytic interstitial pneumonitis, amyloidosis, tracheobronchial papillomatosis, and many others. Regardless of the cause of cysts, patients usually present with either no symptoms, with the cysts discovered on chest imaging for another reason, or with nonspecific symptoms such as cough and shortness of breath [2]. Because of such non-specific presentation, radiographic imaging with CT/positron emission tomography (PET)-CT remains the standard modality to estimate the severity of the disease. The temporal course of the disease process can be assessed based on the duration and symptoms as well as comparison to previous imaging studies, when available. Acute and subacute courses generally suggest infectious or inflammatory disorders while a chronic course is more likely to be due to noninfectious infiltrative processes [3].

Metastasis is an uncommon cause of cystic lung disease. The mechanism of its development remains largely unclear. However, there have been a few mechanisms, proposed to explain the development of these metastatic lesions. One of such mechanisms for the formation of cysts in the lungs is the ball and valve mechanism following endobronchial deposits in small airways. This causes progressive trapping of air and thinning of the nearby lung parenchyma, subsequently forming a cyst. Another mechanism that has been associated with squamous malignancies is liquefaction and expectoration of the central necrotic material, leaving behind an air-containing space. All these proposed mechanisms have been derived from the pathogenesis of lymphangioleiomyomatosis [4].

The most commonly associated malignancies are sarcomas, colorectal carcinomas, and head & neck malignancies [5]. Cystic lung disease in association with pancreatic carcinoma has only been described once in the literature [1]. In the previously reported cases of pulmonary cystic metastases, the primary malignancy, which was sarcoma, was only detected when the patients started showing the non-specific respiratory symptoms that warranted the need of undergoing a CT scan. The presence of these cysts can therefore be considered an indicator of advanced malignancy [6]. Furthermore, atypical pulmonary metastasis has been known to be associated with adenocarcinoma [7]. While the diagnosis of such presentation can be difficult to establish, it has been previously stated in the literature that the presence of irregular but thick-walled cysts on CT scan point toward metastasis as the most likely etiology [8]. The case that we have encountered here is a rare presentation of pancreatic carcinoma in the form of cystic lung disease. We were not able to directly demonstrate the tumor cells in lung tissue as the patient was not fit for bronchoscopic procedures due to high oxygen requirements. However, the temporal course of the development of symptoms, and the radiological pattern of cysts indicate their metastatic origin.

## Conclusions

The presence of bilateral cystic lesions with acute respiratory symptoms in a smoker should create suspicion of cystic metastasis. Serum biomarkers can thus play an important role in diagnosis by pointing toward the primary etiology. However, such presentation isn't encountered till the advanced stages of malignancy and, therefore, the presence of cystic metastasis can be considered as an indicator of extremely poor prognosis. 
